# Morphology, performance and attachment function in *Corynosoma* spp. (Acanthocephala)

**DOI:** 10.1186/s13071-018-3165-1

**Published:** 2018-12-13

**Authors:** Francisco Javier Aznar, Jesús Servando Hernández-Orts, Juan Antonio Raga

**Affiliations:** 1 Instituto Cavanilles de Biodiversidad y Biología Evolutiva, Parque Científico, Universidad de Valencia, Catedrático José Beltrán 2, 46980 Paterna Valencia, España; 2 Centro de Investigación Aplicada y Transferencia Tecnológica en Recursos Marinos Almirante Storni (CIMAS - CCT CONICET - CENPAT), Güemes 1030, 8520 San Antonio Oeste, Río Negro Argentina

**Keywords:** Acanthocephala, Polymorphidae, *Corynosoma*, Attachment, Performance, Muscle, Ecomorphology

## Abstract

**Background:**

Functional inference on the attachment of acanthocephalans has generally been drawn directly from morphology. However, performance of structures is often non-intuitive and context-dependent, thus performance analysis should be included whenever possible to improve functional interpretation. In acanthocephalans, performance analysis of attachment is available only for *Acanthocephalus ranae*, a species that solely relies on the proboscis to attach. Here we compare body morphology and muscle arrangement in 13 species of *Corynosoma*, which use their spiny body as a fundamental holdfast. A basic performance analysis using live cystacanths of two representative species is also provided.

**Methods:**

Adults of 13 *Corynosoma* spp. were obtained from 11 marine mammal species. Specimens were cut and carefully cleaned to examine muscle arrangement through light and scanning electron microscopy. Live cystacanths of *C. australe* and *C. cetaceum* were selected for performance analysis. Video records of evagination-invagination cycles of the proboscis were obtained and analysed with a video editor.

**Results:**

The basic arrangement of proboscis retractors, trunk circular and longitudinal muscles, neck retractors and receptacle retractors, was conserved in all *Corynosoma* species. Interspecific variability was found in the relative development of disk muscles: minimum in *C. enhydri*, maximum in *C. cetaceum*; the distal insertion of the ventral neck retractor: ventro-lateral in *C. cetaceum*, *C. hamannni* and *C. pseudohamanni* and ventral in the other species; and the distal insertion of the receptacle retractors: more proximal in species with a longer hindtrunk. Performance analysis indicated striking similarities to that described for *A. ranae* except that (i) the foretrunk bends ventrally during the evagination-invagination cycles of the proboscis; (ii) disk muscles can flatten the tip of the foretrunk regardless of these cycles; and (iii) the receptacle bends ventrally and is driven to the hindtrunk by coordinated action of receptacle retractors.

**Conclusions:**

Species of *Corynosoma* are able to use up to six holfast mechanisms. Attachment relies on a similar performance to that described for *A. ranae*. However, structural ventral bending of an inflated, spiny foretrunk, with a parallel re-arrangement of foretrunk muscles, have generated unexpected novel functions that make attachment extremely effective in species of *Corynosoma*. Interspecific variability in trunk shape and muscle arrangement grossly correlates with the rheological conditions each species experiences in their microhabitats within the gut of marine mammals.

**Electronic supplementary material:**

The online version of this article (10.1186/s13071-018-3165-1) contains supplementary material, which is available to authorized users.

## Background

From a functional and evolutionary perspective, the morphology of most parasites is largely driven by the need for an effective attachment to their hosts. Acanthocephalans in particular, have developed a proboscis armed with hooks that anchors to the gut of their definitive vertebrate hosts [1]. Many species also use secondary mechanisms that may play an even more prominent role as attachment devices [2, 3].

Functional inferences on the attachment of acanthocephalans have generally been drawn directly from their morphology. For instance, in a series of recent studies, Herlyn & Ehlers [4], Herlyn [5], and Herlyn & Taraschewski [6] provided painstaking descriptions of the muscular apparatus of several acanthocephalan species, and made basic inferences on their function and evolution. However, the performance of any structure, which is the crucial link between its morphology and function, is often non-intuitive and context-dependent [7, 8]. As far as we are aware, there is a single acanthocephalan species for which a complete account of its attachment performance has been carried out. Hammond [9–11] used live detached specimens of *Acanthocephalus ranae* (Schrank, 1788) to describe cycles of evagination-invagination of the proboscis as well as the mechanisms that worms actually use to anchor to the intestinal wall of toads (see Additional file 1: Data S1 and Additional file 2: Figure S1 for a brief description of the morphology, performance and attachment function in *A. ranae*). This approach allowed this author to unveil details of the attachment function that could have easily been overlooked from examination of morphology alone.

**Fig. 1:**
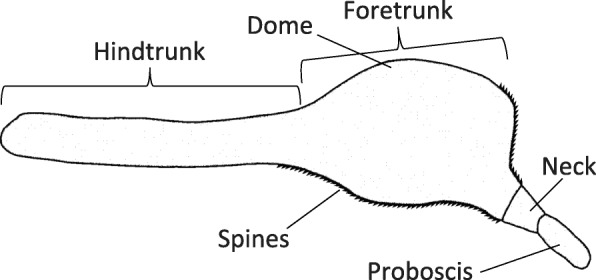
Schematic drawing of the external morphology of *Corynosoma* spp.

Species of *Corynosoma* Lühe, 1904 (Palaeacanthocephala: Polymorphidae) infect a wide array of marine mammal species, and more rarely marine birds, worldwide [12]. The body morphology of this group is peculiar: the foretrunk is inflated and ventrally bent, giving the animals a pipe-shaped appearance. Furthermore, the ventral side of the trunk is covered, to a variable extent, with spines (Fig. 1). Aznar et al. [3] investigated the attachment function of *C. cetaceum* Johnston & Best, 1942 based on a detailed description of external morphology and foretrunk musculature. More recently, Aznar et al. [12] provided a basic description of foretrunk muscles in additional species of *Corynosoma* and proposed a general attachment mechanism for species of this genus, following previous insight from Van Cleave [2]. In essence, species of *Corynosoma* use the flattened, spiny foretrunk as a very efficient device that assists the proboscis to adhere to the gut wall, but are also able to put the ventral hindtrunk into contact with the substratum, reinforcing attachment.

In this paper, we investigate the attachment function in species of *Corynosoma* using a more comprehensive approach. First, we describe the foretrunk musculature in 13 species encompassing the widest morphological variation in a genus with *c.* 31 spp. described [12, 13]. This information is, for the most part, new for all species except *C. cetaceum*. However, it is important to stress from the outset that our approach is functional and, therefore, we will not pay attention to subtle differences in muscle arrangement that are more meaningful in an evolutionary context (see, e.g. [6]). Secondly, we use live cystacanths of two species to explore the link between muscle arrangement and performance. Cystacanths of *Corynosoma* exhibit the same morphology as adults except that they are sexually immature [14], but their anatomy is more visible by transparency. We recorded a range of body movements, including cycles of evagination-invagination of the proboscis, to infer the attachment mechanisms. Finally, we combine morphological and performance data to understand how species of *Corynosoma* actually attach, and to explore the relationship between interspecific morphological variability and microhabitat selection within the host.

## Methods

### Comparison of foretrunk musculature

Adult individuals of 13 *Corynosoma* spp. were obtained from 11 marine mammal species around the world based on specific sampling or requests to museums or particular collections. Details of host identity, locality of collection, and number of specimens examined are shown in Table 1. Most species came from old opportunistic collections and information on specific sampling localities was not available. Specimens had all been collected from stranded or by-caught hosts. Worms were found dead, removed from the intestine or the stomach, washed in saline, and fixed in 70% ethanol at room temperature.

**Table 1:** Collection data for adult specimens of *Corynosoma* spp. examined in this study

Species	Host	No. of worms		Locality
		Males	Females	
*C. australe* Johnston, 1937	*Otaria flavescens* Shaw	10	10	Off Puerto Madryn (Argentina)
*C. bullosum* (Linstow, 1892)	*Mirounga leonina* (L.)	5	5	Antarctica
*C. caspicum* Golvan & Mokhayer, 1973	*Pusa caspica* Gmelin	10	10	Caspian Sea
*C. cetaceum* Johnston & Best, 1942	*Pontoporia blainvillei* (Gervais & d’Orbigny)	10	10	Off Necochea (Argentina)
*C. enhydri* Morozov, 1940	*Enhydra lutris* (L.)	10	10	Off British Columbia (Canada)
*C. hamanni* Linstow, 1892	*Hydrurga leptonyx* (Blainville)	10	10	Antarctica
*C. magdaleni* Montreuil, 1958	*Pusa hispida* Schreber	5	5	Lake Saimaa (Finland)
*C. pseudohamanni* Zdzitowiecki, 1984	*Hydrurga leptonyx* (Blainville)	3	3	Antarctica
*C. semerme* (Forssell, 1904)	*Phoca vitulina* L.	5	5	Off Germany
*C. strumosum* (Rudolphi, 1802)	*Pusa hispida* Schreber	10	10	Off Russia
*C. validum* Van Cleave, 1953	*Odobenus rosmarus* (L.)	10	10	Off Russia
*C. villosum* Van Cleave, 1953	*Eumetopias jubatus* (Schreber)	10	10	Bering Sea
*C. wegeneri* Heinze, 1934	*Pusa hispida* Schreber	10	10	Off Russia

For examination of foretrunk muscles we followed the methodology described in Aznar et al. [3]. Specimens were cut with a razor blade through the transversal or mid-sagittal plane, carefully cleaned to reveal the muscular arrangement, and stained with eosine before examination under a stereomicroscope. Drawings were made with the aid of a drawing tube. Pieces of tegument were also stained with eosin and observed under light microscope (100–400×). To investigate structural details, some specimens were dehydrated through an ethanol series, critical point-dried, and coated with a gold-palladium alloy to a thickness of 250 nm. Specimens were then examined with a Hitachi 4100FE scanning electron microscope operating at 10–20 kV.

### Performance

We adopted the methodology used by Hammond [10] to examine worm movements with a reasonable view of internal structures. A total of 10 cystacanths of *C. australe* were collected from a sample of 42 gutted Argentinean hakes, *Merluccius hubbsi* Marini, captured with fishing lines in the San Matías Gulf, Argentina (40°50'–42°15'S, 63°45'–65°00'W) from June to November 1997. Hake size ranged from 57 to 78 cm (mean ± standard deviation, 67.5 ± 5.7 cm). The fish were air freighted and imported fresh by a Spanish supermarket company. The hake were examined 3 to 4 days after capture. A total of 14 cystacanths of *C. cetaceum* were collected from 5 flounders, *Xystreurys rasile* (Jordan) collected by Argentine hake trawlers in waters of the central Patagonian shelf, Argentina (47°00'–47°19'S, 61°59'–64°25'W) in March 2007. Flounder size ranged from 32.6 to 36.9 cm (34.4 ± 1.7 cm) and were examined fresh 4 to 5 days after capture.

Live cystacanths were removed from fish mesenteries and put in a Petri dish with 0.9% saline. Active worms deployed a range of movements including frequent evagination-invagination cycles of the proboscis apparatus. We recorded worm movements with a Sony DSC-S60 camera connected to a stereomicroscope (20–40×) using transmitted light. Videos were then edited with the open source VLC Media Player 2.0.6.

## Results

### Morphology

For comparative purposes, we follow Hammond [10] for muscle nomenclature. However, when necessary we also provide, in brackets, the terminology used by Aznar et al. [3, 12] to ensure equivalence.

#### Trunk muscles

##### Trunk circular muscles (TCs) [transversal muscles]

Layer of circular muscles lining the trunk wall, being arranged as transversal circular bands. Bands usually single and roughly symmetrical in cylindrical parts, but branching off on the dome (Fig. 1) to cover the additional surface produced by the curvature and inflation of the dorsal foretrunk.

##### Trunk longitudinal muscles (TLs)

**Fig. 2:**
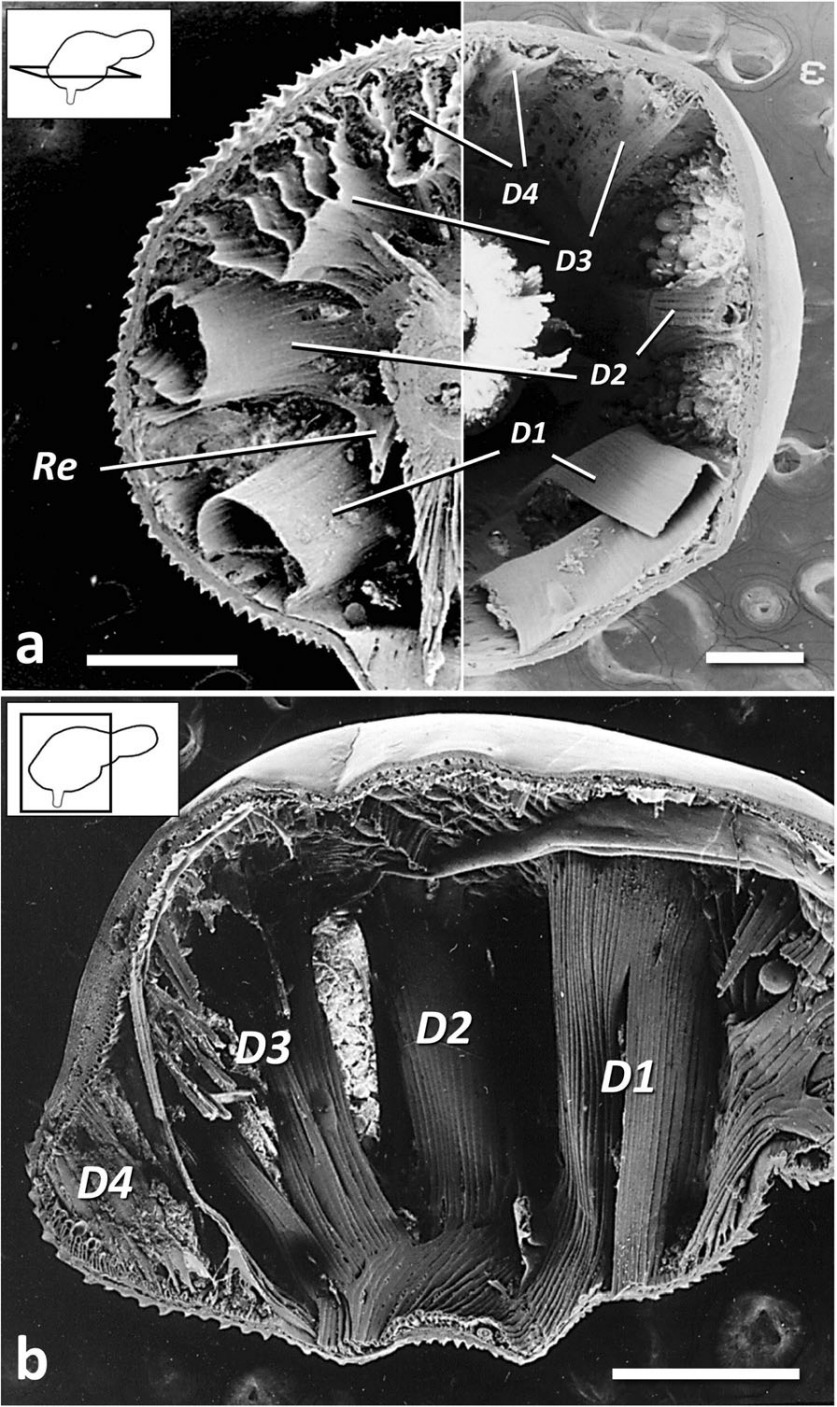
Disk muscles in *Corynosoma* spp. **a** Transversal view of half foretrunk of female *C. cetaceum* (left) and female *C. enhydri* (right). **b** Saggital view of female *C. cetaceum*. *Scale-bars*: 0.5 mm. *Abbreviations*: D1-D4, disk muscles 1-4 (abbreviated as in Aznar et al. [3]); Re, retinaculum

**Fig. 3:**
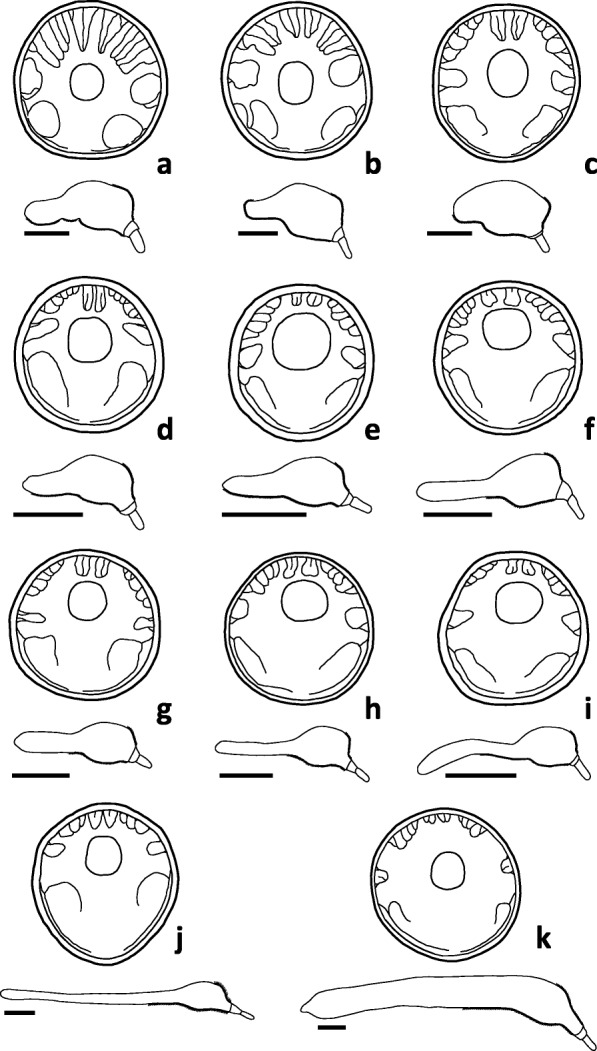
Lateral trunk view and schematic transversal view of disk muscles in females of *Corynosoma* spp. **a** *C. cetaceum*. **b** *C. hamanni* / *C. pseudohamanni*. **c** *C. validum*. **d** *C. australe*. **e** *C. semerme*. **f** *C. caspicum*. **g** *C. villosum*. **h** *C. wegeneri*. **i** *C. strumosum* / *C. magdaleni*. **j** *C. bullosum*. **k** *C. enhydri*. *Scale-bars*: 2 mm

Most of them expanding toward the centre of the disk, leaving lines of contact only with the foretrunk wall, and becoming organized as semi-tubular bundles, collectively named as disk muscles (Ds) [3]. Ds arranged singly or in tightly packed groups (Fig. 2a) with the appearance of “columns” on a sagittal view (Fig. 2b). Four recognizable groups, i.e. D1, D2, D3 and D4 [3] (Figs. 2 and 3).

##### Comments

The basic arrangement in four groups is conserved in all *Corynosoma* species, but the radial development of D3 and D4 is rather variable (Fig. 3), being minimal in *C. enhydri* and maximal in *C. cetaceum* (Fig. 2a). The greater relative development of these muscles appears to be associated with a more centred position of the proboscis coupled with a wider transversal expansion of the proximal half of the disk, such as it is observed in *C. cetaceum*, *C. hamanni* and *C. pseudohamanni*, and to a lesser extent, *C. validum* (Fig. 3). Also, the D1 is arranged as a semi-folded sheet in all species except *C. hamanni*, *C. pseudohamanni* and especially *C. cetaceum*, in which folding progresses to form a nearly closed tube (Fig. 2a).

#### Neck retractor muscles (NRs)

**Fig. 4:**
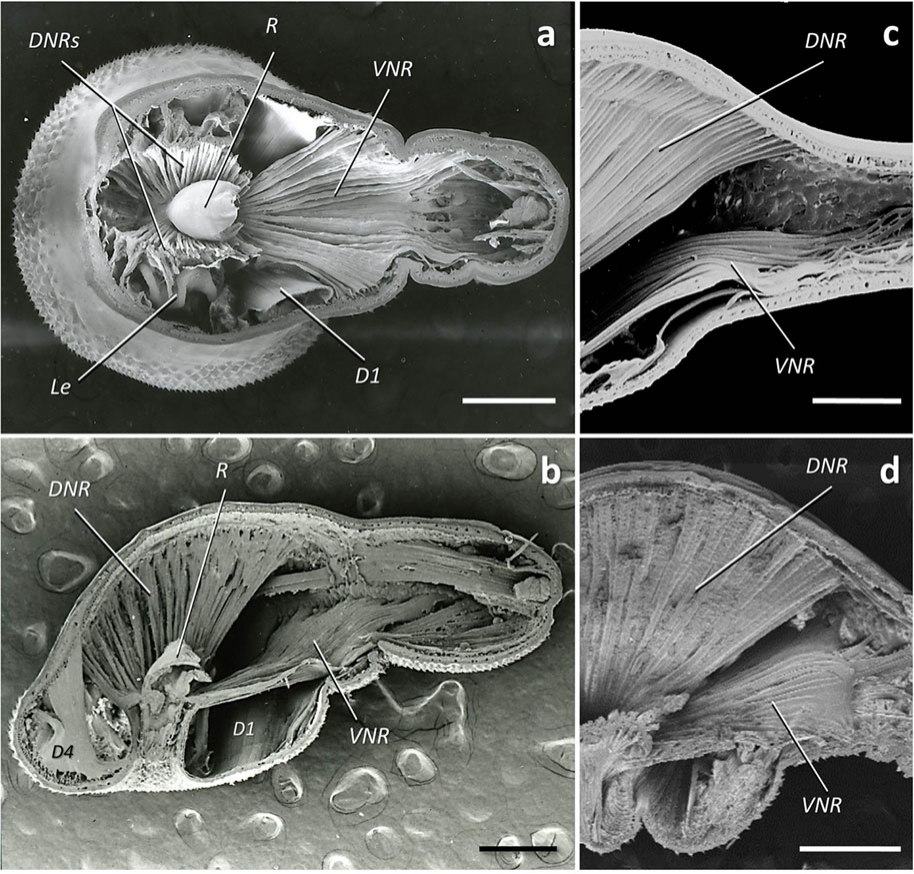
Neck retractor muscles in *Corynosoma* spp. **a** Dorsal view of female *C. cetaceum*. **b** Lateral view of female *C. cetaceum* cut through mid-sagittal plane. Note that the receptacle is cut in both pictures to better view muscle arrangement. **c** Detail of distal insertion of neck retractors in female *C. wegeneri*. **d** Detail of distal insertion of neck retractors in female *C. hamanni*. *Scale-bars*: 0.5 mm. *Abbreviations*: D1, D4, disks muscles 1, 4; DNR(s), dorsal neck retractor(s); Le, lemniscus; R, receptacle; VNR, ventral neck retractor

**Fig. 5:**
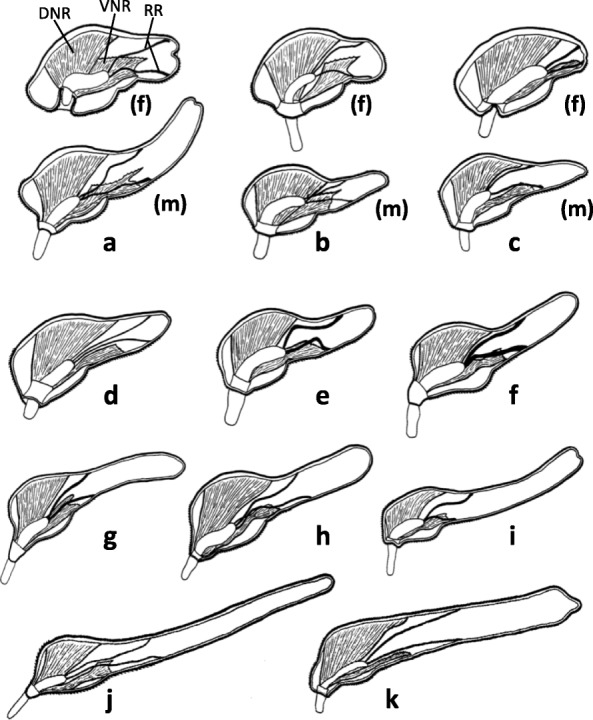
Diagrammatic mid-sagittal view of *Corynosoma* spp. (not to scale), showing arrangement of dorsal neck retractor (DNR), ventral neck retractor (VNR) and receptacle retractors (RR). In species with sexual dimorphism in shape both male (m) and female (f) are shown. **a** *C. cetaceum*. **b** *C. hamanni* and *C. pseudohamanni*. **c** *C. validum*. **d** *C. australe*. **e** *C. semerme*. **f** *C. caspicum*. **g** *C. villosum*. **h** *C. wegeneri*. **i** *C. strumosum* and *C. magdaleni*. **j** *C. bullosum*. **k** *C. enhydri*

With dorsal and ventral bundles. Dorsal portion divided into two large bundles (DNRs) that insert around the neck except its posterior part, fanning out to attach longitudinally along the dorsal dome (Figs. 4 and 5), and also experiencing a substantial transversal expansion (Fig. 4a). Ventral neck retractor (VNR) single, inserting in the posterior portion of the neck, fanning out to attach on the ventral, or ventro-lateral, part of the hindtrunk (Fig. 4a, b). Lemnisci not associated to NRs in any species.

##### Comments

The basic arrangement of DNRs and the VNR is similar in all *Corynosoma* species. However, the distal insertion of VNR can reach far beyond the dome in *C. australe*, *C. enhydri* and males of *C. validum* (Fig. 5). The distal insertion of the VNR is ventral in most species of *Corynosoma*, but the distal attachment experiences a great expansion onto the lateral hindtrunk in *C. hamanni*, *C. pseudohamanni* and especially *C. cetaceum* (Figs. 4 and 5). The lemnisci typically arise in the external side of the DNRs (Fig. 4a). However, in *C. enhydri*, the lemnisci are curved and embrace the posterior part of the DNRs on both sides.

#### Proboscis retractor muscles (PRs)

Several bundles of longitudinal muscles running from the tip of the proboscis to the bottom of the proboscis receptacle. No further, more detailed examination was carried out in any species.

#### Receptacle retractor muscles (RRs)

Double, with thin dorsal and ventral bundles (Fig. 5). Proximal insertion at the tip of the receptacle and distal insertion at roughly the same point of the dorsal (DRR) or ventral (VRR) mid-sagittal plane of the hindtrunk (Fig. 5).

##### Comments

The distal insertion of the RRs is highly variable among species of *Corynosoma*. Five species lack sexual dimorphism in body shape and have a long hindtrunk relative to foretrunk, i.e. *C. bullosum*, *C. enhydri*, *C. magdaleni*, *C. strumosum* and *C. wegeneri*. In these species, except *C. enhydri*, the distal insertion of RRs occurs anterior to mid-hindtrunk (Fig. 5). In *C. enhydri*, and in the species with a medium-sized hindtrunk (i.e. *C. villosum*, *C. caspicum*, *C. semerme* and *C. australe*), the distal insertion of RRs is at the mid-hindtrunk in both sexes (Fig. 5). Finally, 4 species (i.e. *C. cetaceum*, *C. hamanni*, *C. pseudohamanni* and *C. validum*) exhibit clear dimorphism in body shape, with females having a shorter hindtrunk. The distal insertion of RRs is at the mid-hindtrunk (males) or distal hindtrunk (females) (Fig. 5).

### Performance

#### Evagination-invagination of the proboscis

**Fig. 6:**
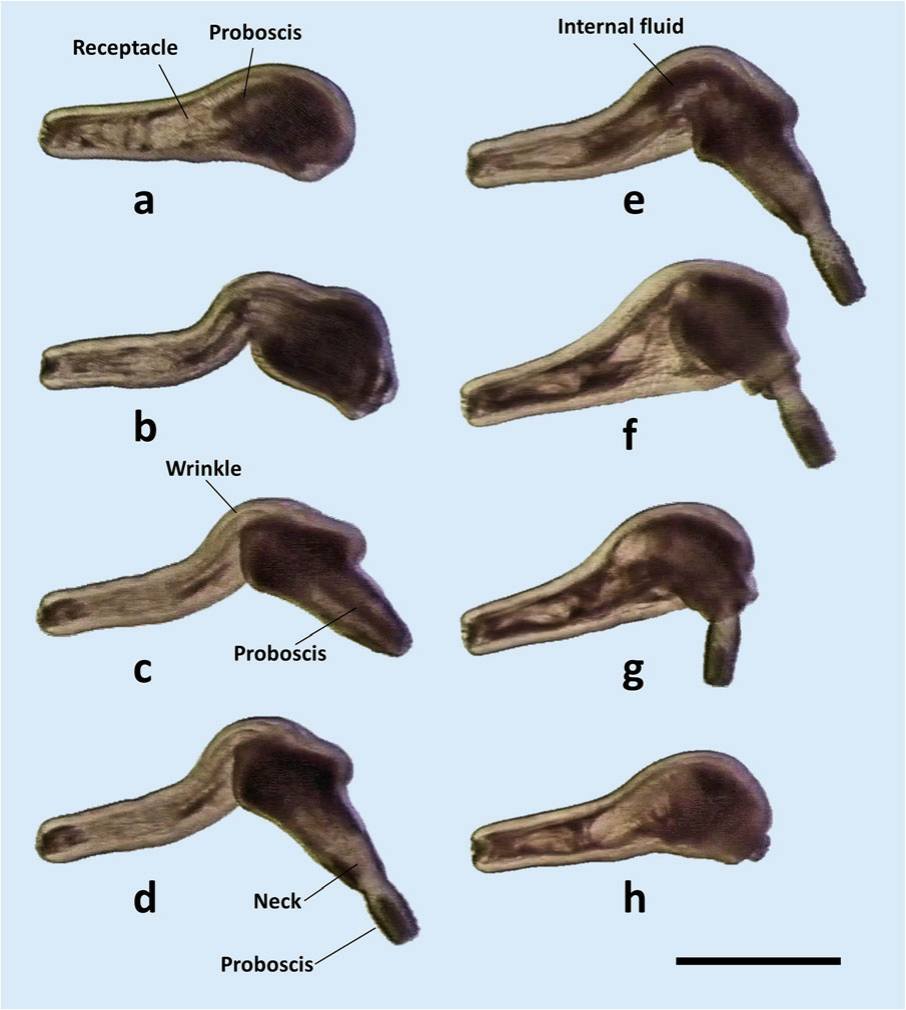
Cycle of evagination-invagination of the proboscis in a male cystacanth of *Corynosoma australe*. Sequence of events have been labelled with letters (**a**-**h**) to ease explanation (see the text for details). *Scale-bar*: 1 mm

**Fig. 7:**
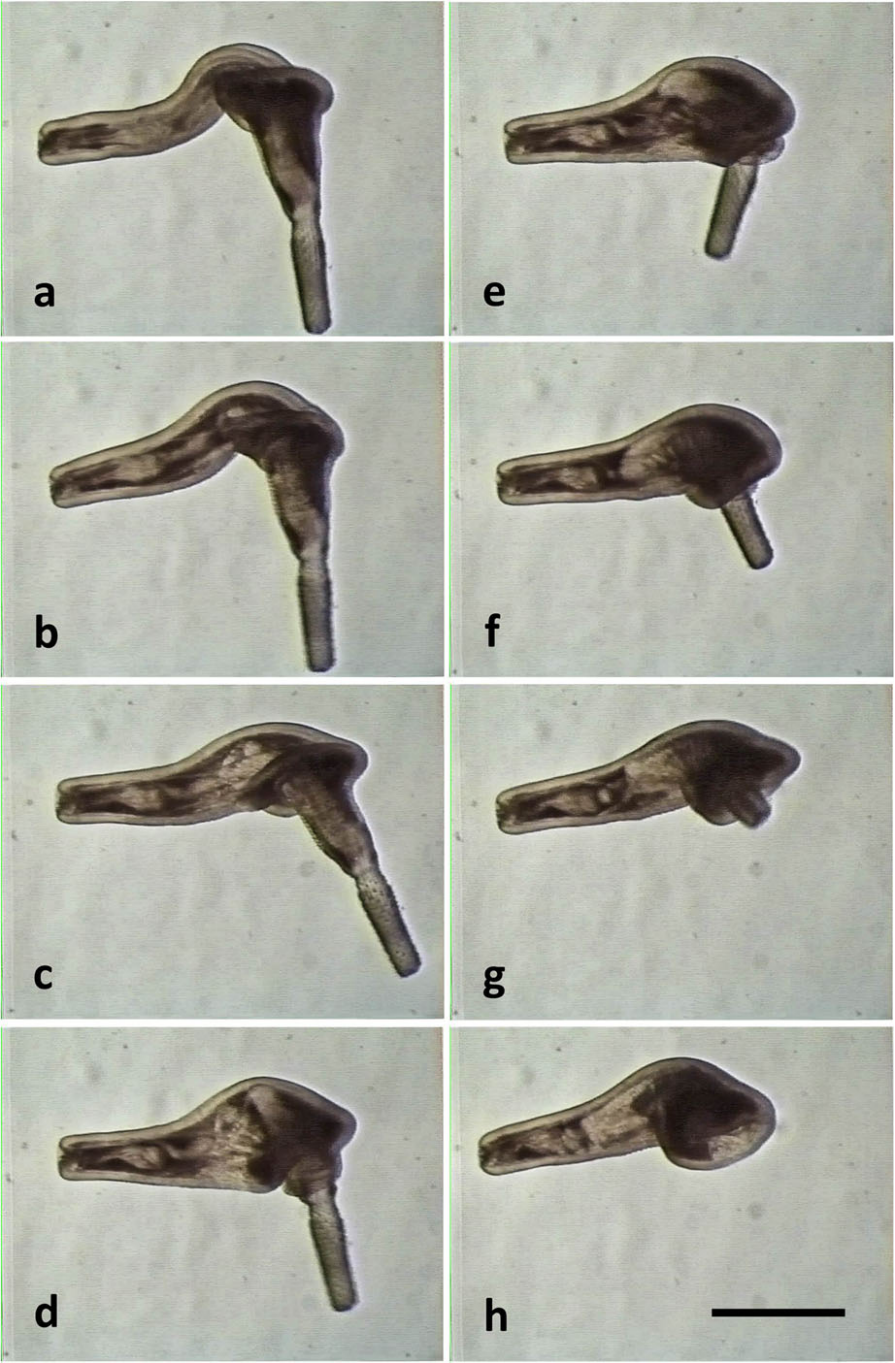
Details of disk formation, proboscis eversion and bending of the proboscis receptacle in a male cystacanth of *Corynosoma australe*. Sequence of events have been labelled with letters (**a**-**h**) to ease explanation (see the text for details). *Scale-bar*: 1 mm

A cycle of evagination-invagination of the proboscis in cystacanths of *C. australe* is shown in Fig. 6. The cycle is similar in *C. cetaceum* (not shown). The cycle starts with the presoma withdrawn within the body cavity, and the proboscis within the proboscis receptacle (Fig. 6a). Then, there is a strong contraction of the TCs on the hindtrunk; contraction is so strong that the hindtrunk tegument becomes longitudinally wrinkled, perhaps pushing the fluid of the lacunar system forwards (Figs. 6b–d and 7a, b). Such contraction provokes: (i) an elongation and reduction in diameter of the hindtrunk (Fig. 6b); (ii) an increase of internal pressure, which squeezes fluid forwards (fluid movement can be observed in Additional file 3: Video S1, Additional file 4: Video S2, Additional file 5: Video S3), pushing the proboscis receptacle forwards and forcing the presoma to unfold (Figs. 6c, d and 7b, c); and (iii) a downward bending of the foretrunk as a passive effect of an increased hydrostatic pressure on the inflated, ventrally curved foretrunk (Figs. 6b and 7a). Unfolding requires that the NRs and RRs are relaxed, but the Ds may or may not contract to flatten the foretrunk, forming the disk (Fig. 7, Additional file 3: Video S1, Additional file 4: Video S2, Additional file 5: Video S3). During the unfolding of the proboscis apparatus, the circular muscles of the receptacle also contract, evaginating, fully or partially, the proboscis (Fig. 6d, Additional file 3: Video S1, Additional file 4: Video S2, Additional file 5: Video S3).

Once the proboscis is everted, the TCs relax, and the hyper-pressurized fluid accumulated in the foretrunk moves passively backwards, reaching even the posterior tip of the hindtrunk (Fig. 6e, f). The NRs then contract, invaginating the proboscis apparatus; the receptacle is driven to an inner position within the hindtrunk by the coordinated contraction of dorsal and ventral RRs, which ventrally bend the receptacle once it contacts the dome (Figs. 6f-h and 7d-f). The proboscis can remain everted or be invaginated by the contraction of the PRs (Additional file 3: Video S1, Additional file 4: Video S2, Additional file 5: Video S3).

#### Disk formation and hindtrunk movement

**Fig. 8:**
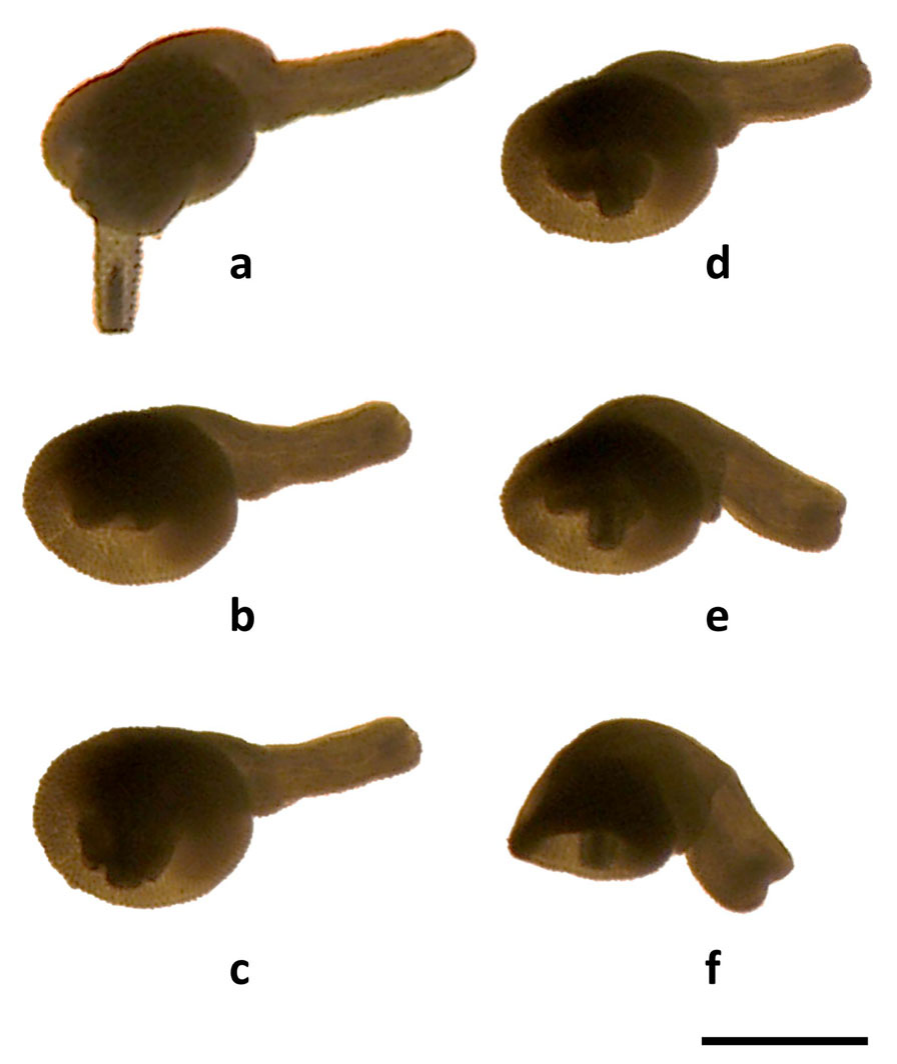
Sequence of hindtrunk downward movement in a female cystacanth of *Corynosoma cetaceum*. Sequence of events have been labelled with letters (**a**-**f**) to ease explanation (see the text for details). *Scale-bar*: 1 mm


The disk is formed by the contraction of Ds, which can flatten the tip of the foretrunk independently of the evagination-invagination cycle of the proboscis (Fig. 7, Additional file 3: Video S1, Additional file 4: Video S2, Additional file 5: Video S3). Deep contraction of the inner portion of Ds results in the formation of a circular inward fold of tegument (Fig. 8). In contrast, the TCs of the foretrunk can generate a tubular invagination of the foretrunk tip when the presoma is withdrawn within the trunk (Fig. 7f-h, Additional file 3: Video S1, Additional file 4: Video S2, Additional file 5: Video S3). The local, antagonistic action of Ds and TCs, mediated by the hydrostatic skeleton, can generate an impressive variety of movements and shapes of the disk (Additional file 3: Video S1, Additional file 4: Video S2, Additional file 5: Video S3 and Additional file 6: Video S4).

**Fig. 9:**
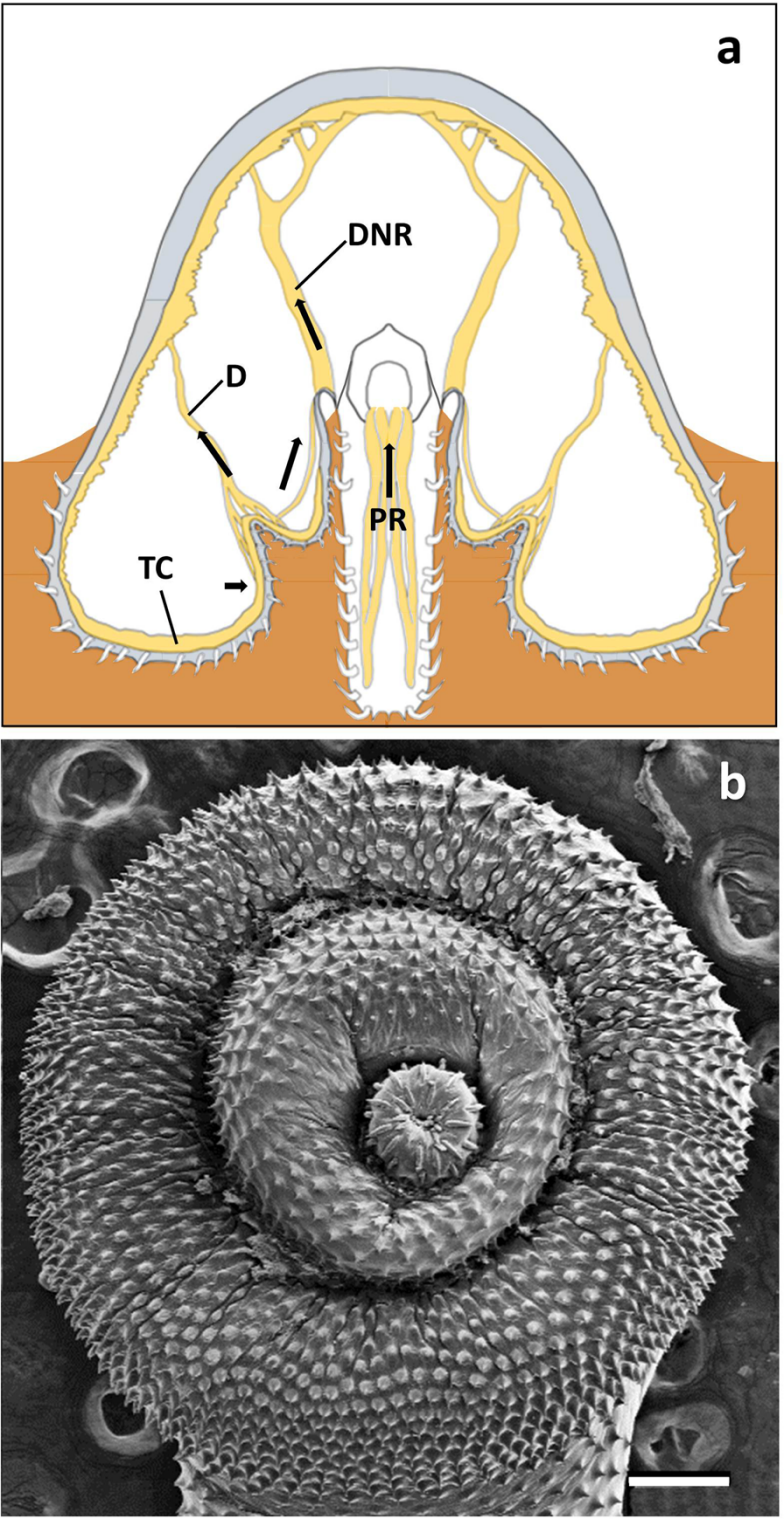
Attachment mechanisms in *Corynosoma* spp. **a** Fronto-transversal view of foretrunk of a female *C. cetaceum*, showing major foretrunk muscles and lines of contraction (re-drawn from Aznar et al. [3]). **b** Ventral view of the disk in a female *C. hamanni*. *Abbreviations*: D, disk muscle; DNR, dorsal neck retractor; PR, proboscis retractor; TC, trunk circular muscles

Additional file 6:**Video S4**. Ventral folding of the foretrunk in a female cystacanth of *Corynosoma cetaceum* collected from the flounder, *Xystreurys rasile*. For scale-bar see Fig. 9. (MP4 633 kb)


In *C. australe*, the ventral (spiny) side of the hindtrunk becomes aligned with the disk during the invagination-evagination cycle of the proboscis (Figs. 6 and 7). However, *C. cetaceum* can move the hindtrunk downwards by a strong contraction of the VNR. This contraction shortens and ventrally tilts the hindtrunk, producing a deep fold on its ventral side (Fig. 8, Additional file 6: Video S4).

## Discussion

### Holdfast mechanisms in *Corynosoma* spp.

According to the above evidence, we suggest that the attachment of *Corynosoma* spp. to their hosts relies on the interplay of several mechanisms (Fig. 9). First, the proboscis can be withdrawn and anchored to the gut wall. In *A. ranae*, Hammond [11] noted that the proboscis is fully everted only when the animal firstly engages in the mucosa, but not when the worm is fully attached. Apparently, full evagination helps prevent the newly recruited worms from being expelled when they attempt first (re)attachment. In *C. australe* and *C. cetaceum* we observed both partial and complete eversion of the proboscis during cycles of invagination-evagination. Likewise, in fully attached individuals of *Corynosoma* spp. there are observations of worms with the proboscis fully [15] or partially [16] everted. It is therefore possible that the relatively long proboscis typical of *Corynosoma* spp. (see references in Aznar et al. [12]) functions as a versatile attachment structure in hosts with intense peristalsis [14].

Once the proboscis penetrates the gut wall and engages in the tissue, contraction of the DNRs can pull the foretrunk against the substratum. This mechanism is similar to that described for *A. ranae* [10, 11] but it is far more effective in *Corynosoma*. The structural bending of the foretrunk allows a spectacular development of the DNRs, which can generate a strong reaction force perpendicular to the substratum [3, 12]. Also, the tip of the foretrunk is flattened, thus providing a greater surface of contact with the gut wall, with spines reinforcing adherence (Fig. 9b). Moreover, the host’s tissue is so strongly pulled upwards by the DNRs that a crater is frequently formed during deep attachment [2, 16]. This elevation of the substratum often traps host tissue between the hooks of the proboscis and the spines on the opposite disk wall, “...as though staples had been applied” on it [2] (Fig. 9b). The crater also increases resistance to horizontal drag and can be embraced by the circular muscles of the foretrunk to reinforce adherence (Additional file 3: Video S1, Additional file 4: Video S2, Additional file 5: Video S3 and Additional file 6: Video S4).

Disk muscles, when contracted strongly, can also create a second inward fold that also ‘sucks’ and traps host tissue (Fig. 9b). If such a process is combined with contraction of circular muscles in the hindtrunk, and relaxation in the foretrunk, the net effect should also be an expansion of the disk border. In deeply attached worms, this expansion would produce a ‘wedge’ effect against the gut wall because the disk is literally buried in the mucosa ([16]; F.J. Aznar, unpublished observations). Not surprisingly, disk spines are larger on the border than anywhere else on the disk [17].

Finally, structural bending of the trunk in *Corynosoma* spp. also allows the hindtrunk to contact the substratum in fully attached worms. In fact, all species have developed a ventral field of spines of variable extension that further increases worm’s adherence [3, 12]. Structural bending also brings about a re-arrangement of the ventral bundles of the neck retractors, i.e. the VNR, which could generate up-and-down movements of the hindtrunk depending on the relative position of the proximal and distal insertion. If the proximal insertion is lower (e.g. when the neck is at least partly evaginated) VNR contraction would generate an upward torque; if it is at the same level (e.g. when the neck is deeply invaginated) contraction would produce a downward torque (Additional file 6: Video S4). Upward and downward movements of the hindtrunk are potentially related with at least two functions, i.e. copula, and attachment of the hindtrunk, respectively ([3, 17, 18], see below).

### Interspecific variability

The 13 species of *Corynosoma* included in this study encompass the widest variation in body morphology found within this genus. Trunk size differed by an order of magnitude between the smallest (*C. australe* and *C. semerme*) and the largest (*C. bullosum* and *C. enhydri*) species [17] (Fig. 3). Furthermore, there were species with very stout bodies and a short hindtrunk (e.g. females of *C. cetaceum*, *C. hamanni*, *C. pseudohamanni* and especially *C. validum*) and species with slender bodies and a long hindtrunk (e.g. *C. strumosum* and *C. bullosum*). Regardless of this variation, the basic arrangement of foretrunk muscles appeared to be conserved in all species and, at the coarse level of analysis we carried out, interspecific differences were found only in three elements with potential functional significance, i.e. the distal insertion of RRs, the transversal expansion of Ds, and the lateral expansion of the VNR. Of course, a more detailed comparison of muscular anatomy (see, e.g. [6]) could reveal further and more subtle interspecific differences in performance and function.

Differences in the distal insertion of RRs are likely related to the role of these muscles in driving the receptacle to a precise position within the hindtrunk when the proboscis apparatus retracts. The receptacle must be bent backwards, requiring a coordinated contraction of the RRs. Apparently, this is possible if the distal insertion of these muscles occurs at a specific point from the dome (Fig. 5, Additional file 5: Video S3 and Additional file 6: Video S4). Therefore, the distal insertion of the RRs should be found at variable points depending on the length of the hindtrunk. This is nicely illustrated by species with sexual dimorphism in body shape: males with a longer hindtrunk than their females exhibit a more anteriad insertion of the RRs (Fig. 5).

There was also a variable degree of transversal development of Ds, especially D3 and D4, being minimal in *C. enhydri* and maximal in *C. cetaceum*. A greater development of Ds allows the worms to flatten a higher portion of the foretrunk, but also moves the neck and proboscis onto a more centred position on the disk (Fig. 3). Interestingly, in *C. cetaceum*, *C. hamanni* and *C. pseudohamanni*, the inward secondary folding that is generated by the Ds results in a complete ring (Fig. 9b; see also figure 1a in Ionita et al. [19]), whilst in species with a more eccentric placement of the neck, only a posterior semi-circular fold can be formed due to space limitation for D3 and D4 (see figure 1c, d in Ionita et al. [19]).

*Corynosoma cetaceum*, *C. hamanni* and *C. pseudohamanni* also suffer significant lateral expansion of the VNR. At least in females of *C. cetaceum*, the VNR assists the animal in performing a downward movement to attach a relatively thick and short hindtrunk [3, 17, 18]. During the process, the hindtrunk somewhat shortens due to ventral folding, and the whole ventral spiny surface contacts the substratum ([17], Additional file 6: Video S4). In *C. hamanni* and *C. pseudohamanni*, the hindtrunk is also short and wholly covered by spines, thus one could postulate a similar performance of the VNR. However, why the VNR is less developed in other species is intriguing, particularly in the case of *C. validum* whose females exhibit a ball-like body shape [20]. In species with a very small body size and a fully-spined hindtrunk, i.e. *C. australe* and *C. semerme* (see also *C. obtuscens* in plate 1 from Van Cleave [20]), the hindtrunk seems able to become horizontally aligned with the disk without the need of a downward movement (Additional file 3: Video S1, Additional file 4: Video S2, Additional file 5: Video S3). In species with a slender hindtrunk, the ventral field of hindtrunk spines hardly reaches the anterior half (Fig. 3, and references in Aznar et al. [12]), raising the question of how they use these spines.

### Ecomorphological patterns

The interspecific variability in body morphology and muscle arrangement in *Corynosoma* spp. should be related, at least in part, with the specific microhabitat conditions each species experiences. Unfortunately, the physical conditions prevailing inside the gut regions of marine mammals are currently unknown and are unlikely to be elucidated in the near future. Moreover, we lack quantitative data on the gut distribution for most *Corynosoma* species. However, it is possible to establish coarse correlations between the morphology of *Corynosoma* spp. and the putative physical conditions of the microhabitats each species occupies based on a great deal of recent data on the physical processes of mammalian digestion [21].

The macroparasites attached to the gut of mammals must withstand shear forces generated by three processes, i.e. (i) mobility of gut contents; (ii) mobility of the protective mucine layer; and (iii) muscle contraction in the small intestine that causes the villi to bunch together, driving out recently secreted masses of mucin, which act in a jet-like manner [21–23]. Changes in shear forces throughout the gut depend on the pseudoplasticity and viscoelasticity of both gut contents and mucin. To date, little information on the changes of physical properties of food contents is available. In the stomach of carnivorous mammals, strong peristaltic waves move ingested food toward the narrowed pylorus so that liquids and small particles (up to 2 mm) continuously flow into the duodenum, but most semi-digested food is squirted back; such retro-propulsion crush and grinds the digesta [22, 24, 25]. The fluid chyme that is sieved through the pylorus is propelled forward by peristaltic waves, but it is also subject to local mixing movements associated with wall segmentation of the small intestine [22, 23]. Absorption makes the chyme progressively change from a semi-liquid state with particulate matter that flows quickly in the duodenum, to a semi-solid viscoelastic material that flows slowly in the terminal ileum [25, 26]. In the large intestine, segmentation and propulsion of increasingly dense contents continue [22]. From a rheological point of view, the digesta flows throughout the gut under a virtual laminar flow, i.e. at low Reynold numbers [25, 26] where frictional drag predominates, being stronger at increasing viscosity and/or propulsion force [27].

To our knowledge, quantitative accounts on the gut distribution of *Corynosoma* spp. are available for just five species. *Corynosoma cetaceum* is exceptional among acanthocephalans in that it favours the antral part of the stomach [18], where the strongest forward-backward propulsion of semi-solid food presumably occurs. Being exposed to the strongest frictional drag, *C. cetaceum* exhibits not only the greatest development of Ds and VNR, but also the largest body spines of all *Corynosoma* species examined thus far [17]. As an interesting twist, females of *C. cetaceum* also have shorter bodies than males, contrary to most acanthocephalans [28], and are able to deeply fold the hindtrunk thanks to VNR contraction [29]. These features additionally reduce frictional drag, which helps females withstand the harsh microhabitat conditions in the stomach longer than males [14]. *Corynosoma strumosum* and *C. magdaleni* favour the jejunum and proximal ileum [30–32]. Rheological conditions in this microhabitat are expected to be more benign because peristaltism is less intense and the digesta more fluid than in the stomach. Additionally, flow is unidirectional and more predictable (note, however, that mucin masses associated with grouping of villi may generate additional drag). Indeed, *C. strumosum* and *C. magdaleni* are long and slender, with a modest development of Ds and VNR and just a reduced field of small spines on the ventral hindtrunk [17]. Finally, *C. australe* and *C. semerme* favour the terminal ileum and large intestine, respectively [30, 33]. Stronger directional drag is expected in these microhabitats because this is where digesta becomes semi-solid and viscous. Apparently, both species have a reduced body size to minimise exposure to frictional drag, but also cover the whole hindtrunk with long spines relative to body size to withstand it.

## Conclusions

*Acanthocephalus ranae* is the only acanthocephalan species for which the attachment mechanism was hitherto described based on detailed observations of muscle arrangement and performance of live worms. The new evidence obtained in this study for 13 *Corynosoma* spp. indicates that their pipe-shaped body, which results from the ventral bending of an inflated foretrunk, brings about significant re-arrangements of foretrunk muscles. These changes, coupled with the possession of trunk spines, significantly improve the basic attachment performance described for *A. ranae*. This neat functional comparison, however, does not necessarily bear direct evolutionary implications. There is a spectacular variability of body shapes in the family Polymorphidae (hence its name), with corresponding differences of attachment function in, e.g., species of *Corynosoma*, *Bolbosoma* Porta, 1908 or *Profilicollis* Meyer, 1931 (see [2]). In particular, the presence of disk muscles, or the separation of two dorsal bundles and a ventral bundle, in neck retractors, are probably a plesiomorphic condition, at least in the clade of the Polymorphidae that contains *Corynosoma* spp. (see [12, 34]). Only a thorough analysis of anatomy and performance of acanthocephalan species within a phylogenetic framework can shed light on the evolution of attachment mechanisms. The comparison between *A. ranae* and *Corynosoma* spp., or between species of *Corynosoma* themselves, also illustrates how changes in attachment performance are driven by the rheological conditions each species experiences in their microhabitats. Species of *Corynosoma* infect carnivorous mammals with strong peristalsis and effective attachment mechanisms are therefore required to hold them in the gut [14]. Furthermore, there seems to be a coarse but clear correspondence between the efficiency of holdfast mechanisms and the specific microhabitat each species favours. Future research should provide more data on the distribution and attachment of species, as well as on the physical conditions within the gut of marine mammals.

## Additional files

Additional file 1: **Data S1.** Morphology, performance and attachment function in *Acanthocephalus ranae*. (PDF 64 kb)

Additional file 2: **Figure S1.** Proboscis evagination mechanism in the acanthocephalan *Acanthocephalus ranae* (see text for details). *Abbreviations*: L, lemnisci; N, neck; NR, neck retractors; P, proboscis; PR, proboscis retractor; R, proboscis receptacle; RR, receptacle retractor. Adapted from [10] with permission of the Company of Biologists, Ltd. (PDF 85 kb)

Additional file 3: **Video S1.** Examples of evagination-invagination cycles in cystacanths of *Corynosoma australe* collected from the Argentine hake, *Merluccius hubbsi*. For scale-bar see Figs. 7 and 8. (MP4 2770 kb)

Additional file 4: **Video S2.** Examples of evagination-invagination cycles in cystacanths of *Corynosoma australe* collected from the Argentine hake, *Merluccius hubbsi*. For scale-bar see Figs. 7 and 8. (MP4 1919 kb)

Additional file 5: **Video S3.** Examples of evagination-invagination cycles in cystacanths of *Corynosoma australe* collected from the Argentine hake, *Merluccius hubbsi*. For scale-bar see Figs. 7 and 8. (MP4 14305 kb)

## Abbreviations

DNR: Dorsal neck retractor muscle
DRR: Dorsal receptacle retractor muscle
Ds: Disk muscles
NR: Neck retractor muscles
PR: Proboscis retractor muscle
RR: Receptacle retractor muscle
TC: Trunk circular muscles
TL: Trunk longitudinal muscles
VNR: Ventral neck retractor muscle
VRR: Ventral receptacle retractor muscle
