# Supplementary material for: Morphology, performance and attachment function in Corynosoma spp. (Acanthocephala)
Source: Parasit Vectors. 2018 Dec 13;11:633. doi: 10.1186/s13071-018-3165-1 (PMC6293589; doi:10.1186/s13071-018-3165-1)
Supplement: Supplementary file 2 — Data S1. Morphology, performance and attachment function in Acanthocephalus ranae. (PDF 64 kb) [file 13071_2018_3165_MOESM1_ESM.pdf]

Additional file 1: Data S1 Morphology, performance and attachment function in *Acantocephalus ranae*

It is important to briefly remind the morphology and performance of the only species previously dealt with, namely *A. ranae*, to unveil details of its attachment function. This will serve as a basis for a comparison with the results from the present study (for further details, see [9–11] in the present paper).

The body of *A. ranae* contains several hydrostatic skeletons (Additional files 2: figure S2). The wall of the proboscis contains the lacunar system, which is made up of interconnected fluid-filled spaces. The proboscis is connected with a proboscis receptacle, both enclosing a fluid-filled cavity. The proboscis receptacle is a double-walled muscular sac with clockwise spiral fibres. A proboscis retractor muscle (PR) runs from the tip of the proboscis to the bottom of the receptacle, and a receptacle retractor muscle (RR) stems externally from the base of the receptacle and laterally inserts to the trunk wall. The neck is connected with the lemnisci, which originate from the inner layers of the neck wall. A partition separates the wall of the neck and the proboscis from that of the trunk. A conical sheet of neck retractor muscles (NRs) stems from the base of the neck and inserts distally on the trunk, also investing the lemnisci completely. The trunk, which is non-spined, represents a third fluid-filled cavity the wall of which is bounded internally by an inner layer of circular muscles (TCs) and an outer layer of longitudinal muscles (TLs).

In detached individuals of *A. ranae*, regular cycles of evagination-invagination of the proboscis occur (Additional files 2: figure S2). When the PR, RR, NRs and TLs are contracted, the proboscis is invaginated and the entire proboscis apparatus is withdrawn within the trunk cavity; an intucking of the foretrunk is formed. When the TCs contract and the TLs relax, the trunk becomes elongated (up to 40%) and reduced in diameter; antagonism is mediated by the hydrostatic skeleton of the trunk. The proboscis apparatus is forced forwards, stretching the RR, the NR and the lemnisci. As foretrunk elongation is complete, the spiral muscle sheath of the receptacle contracts and the PR relaxes, forcing the proboscis out by hydrostatic pressure. Evagination is powerful due to the reduction in volume of the receptacle, which displaces liquid towards the proboscis. The NRs then contract and the TCs partially relax. As a

consequence, the proboscis apparatus is drawn inward, with part of the foretrunk wall, whereas the proboscis remains evaginated. As the NRs contract, they squeeze the lemnisci, forcing its fluids towards the lacunar spaces of the proboscis wall.

Acanthocephalus ranae is attached by projecting the proboscis apparatus towards the intestine wall and sequentially everting the proboscis. For a successful attachment, the basal hooks of the proboscis must engage firmly in the intestinal tissue and hold the worm in place; in this way, the proboscis can penetrate further as it continues evagination. However, mechanic resistance of intestinal tissue typically prevents an entire eversion of the proboscis. Once proboscis attachment is firm, the NRs contract. As a result, the foretrunk is pulled against the intestinal wall, and the lemnisci are squeezed provoking a swelling of the proboscis wall, thus a tighter attachment. A strong contraction of the NRs may also bring about a partial retraction of the proboscis apparatus, pulling a plug of intestinal tissue into the worm.
