# Supplementary figures and images for: Morphology, performance and attachment function in Corynosoma spp. (Acanthocephala)
Source: Parasit Vectors. 2018 Dec 13;11:633. doi: 10.1186/s13071-018-3165-1 (PMC6293589; doi:10.1186/s13071-018-3165-1)

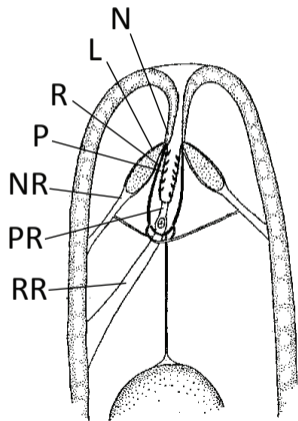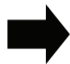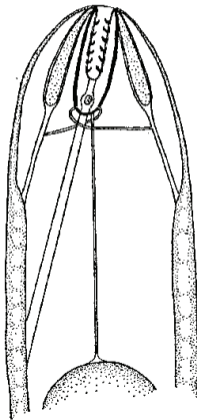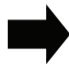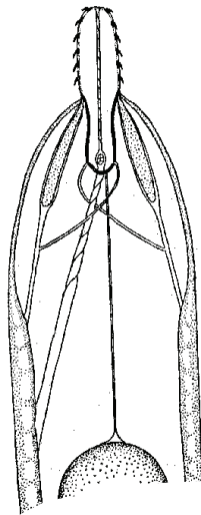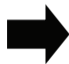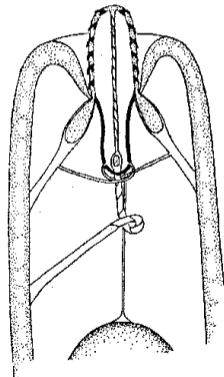

Supplement: Supplementary file 3 — Figure S1. Proboscis evagination mechanism in the acanthocephalan Acanthocephalus ranae (see text for details). Abbreviations: L, lemnisci; N, neck; NR, neck retractors; P, proboscis; PR, proboscis retractor; R, proboscis receptacle; RR, receptacle retractor. Adapted from [10] with permission of the Company of Biologists, Ltd. (PDF 85 kb) [file 13071_2018_3165_MOESM2_ESM.pdf]
